# How is suicide risk assessed in healthcare settings in the UK? A systematic scoping review

**DOI:** 10.1371/journal.pone.0280789

**Published:** 2023-02-02

**Authors:** Sophia E. Fedorowicz, Robert C. Dempsey, Naomi Ellis, Elliott Phillips, Christopher Gidlow

**Affiliations:** 1 Centre for Health and Development, Staffordshire University, Stoke-on-Trent, Staffordshire, United Kingdom; 2 Faculty of Health and Education, Department of Psychology, Manchester Metropolitan University, Manchester, United Kingdom; Xiamen University - Malaysia Campus: Xiamen University - Malaysia, MALAYSIA

## Abstract

A high proportion of people contact healthcare services in the 12 months prior to death by suicide. Identifying people at high-risk for suicide is therefore a key concern for healthcare services. Whilst there is extensive research on the validity and reliability of suicide risk assessment tools, there remains a lack of understanding of how suicide risk assessments are conducted by healthcare staff in practice. This scoping review examined the literature on how suicide risk assessments are conducted and experienced by healthcare practitioners, patients, carers, relatives, and friends of people who have died by suicide in the UK. Literature searches were conducted on key databases using a pre-defined search strategy pre-registered with the Open Science Framework and following the PRISMA extension for scoping reviews guidelines. Eligible for inclusion were original research, written in English, exploring how suicide risk is assessed in the UK, related to administering or undergoing risk assessment for suicide, key concepts relating to those experiences, or directly exploring the experiences of administering or undergoing assessment. Eighteen studies were included in the final sample. Information was charted including study setting and design, sampling strategy, sample characteristics, and findings. A narrative account of the literature is provided. There was considerable variation regarding how suicide risk assessments are conducted in practice. There was evidence of a lack of risk assessment training, low awareness of suicide prevention guidance, and a lack of evidence relating to patient perspectives of suicide risk assessments. Increased inclusion of patient perspectives of suicide risk assessment is needed to gain understanding of how the process can be improved. Limited time and difficulty in starting an open discussion about suicide with patients were noted as barriers to successful assessment. Implications for practice are discussed.

## Introduction

Suicide is a global public health priority with approximately 700,000 deaths by suicide recorded each year across the world [1]. Reducing rates of suicide, and identifying individuals at a heightened risk for suicide, remains a priority for public health practitioners, healthcare professionals, and local and national governance. Reducing suicide mortality by one third is one of the United Nations’ sustainable development goals for 2030 (target 3.4.2.) [2]. Healthcare services provide opportunities for intervention based on identifying those most at-risk of death by suicide. Indeed, there is evidence that in the 12 months prior to suicide, 87% of individuals are in contact with general practice services and one third are in contact with mental health services [3,4]. There is also evidence that help-seeking escalates in the weeks before death, with general practice being the most common last point of contact [5]. Identifying those at highest risk for suicide when they come into contact with healthcare services is crucial.

Suicide risk assessments (SRA) carried out by healthcare practitioners often take the form of psychometric scales, such as the SAD PERSONS scale [6], in order to determine whether a person is at high risk of taking their life and if suicide prevention measures are necessary. Such SRA tools have a number of limitations, including being time consuming to administer and having low levels of accuracy in predicting suicide [7]. Indeed, Carter et al (2018) found the positive predictive value of such risk assessments to be less than 20% [8], and other studies have found that a substantial fraction of patients who died by suicide were considered to be at low risk [9]. Such suicide risk stratification can be informed by a wide range of risk factors, often relying on the identification of depressive feelings in a patient as this is a known risk factor for suicide. However, depression is a common mental health problem that affects more than 264 million people [10] and the presence of depression does not guarantee suicidality. Therefore, questions such as ‘are you feeling depressed?’, which are commonly found in SRA tools, are not useful for healthcare practitioners in determining suicide risk [11]. Furthermore, the UK National Institute for Health and Care Excellence (NICE) guidance aims to reduce the reliance on risk stratification by encouraging assessments that take into account a person’s safety and needs [12]. SRA tools are also not immune to bias as the interpretation of risk factors by practitioners may vary depending on the practitioner’s age and gender, patient age, and whether it is a doctor or a nurse conducting the assessment amongst other characteristics [13,14].

The UK NICE guidelines state that risk assessment tools and scale should not be used to predict future self-harm or to determine who should or should not be offered treatment or discharged. NICE emphasise that healthcare practitioners should focus the assessments on the person’s individual needs and how to support their psychological and physical safety both immediately and in the long term. The assessment process should treat the person with respect, dignity and compassion, with an awareness of cultural sensitivity [12]. Notwithstanding the issues related to reliability or appeals for caution from best practice guidelines, SRA tools continue to be used across the UK with considerable variation between and within NHS services, including the usage of non-validated and locally developed tools [15,16]. There also remains limited guidance for healthcare practitioners on how to assess patients’ suicide risk. The way a patient is asked about suicide, regardless of whether a tool has been used to assess risk, can influence the response that patient gives, inevitably impacting on the outcome of the assessment [17]. This is especially important because evidence suggests healthcare practitioners in the UK may be reluctant to ask patients about suicide because of a lack of confidence in how to respond in a sensitive manner when discussing suicidality-related experiences [18].

Understanding how SRAs are conducted and experienced by healthcare practitioners and patients, rather than the statistical reliability of the tools themselves, could alleviate some of the difficulty practitioners in the UK experience when doing these assessments and improve the patient experience [18]. In addition, healthcare and public health systems vary between countries resulting in different outcomes for people accessing mental health support and varying factors influencing which SRA tools are used and how [19,20]. Therefore, this review aimed to examine the extent and range of evidence relating to how SRAs are conducted and experienced in the UK by healthcare practitioners, patients, carers, relatives and friends of people who have died by suicide. A scoping review was considered appropriate to identify the available evidence, key factors related to SRAs and to identify knowledge gaps [21,22].

## Methods

### Search strategy

The authors followed the PRISMA extension for scoping reviews guidance in developing the review protocol and conducting the database searches [23]. The protocol for this scoping review was registered on the 18^th^ November 2019 with the Open Science Framework [24]. Literature searches were conducted in November and December 2019, and a top-up search was conducted in January 2022 using the following online databases: MEDLINE, CINAHL, PsycARTICLES, Cochrane Library, Science Direct, Scopus, PubMed, ProQuest Nursing, Allied Health Database, Open Grey, and The Grey Literature Report Database. There were no parameters placed on the database searches, except for the January 2022 top-up search where parameters were placed to ensure only publications from between January 2020 and January 2022 were screened (fields searched were Title/Abstract). For ease of reading, both searches are combined in the following synthesis. For complete details of the search, screening, and data extraction, data is available via The Open Science Framework (https://osf.io/5W8ZT/) [24].

Arksey & O’Malley [22] suggest that broad keywords and search terms should be adopted that enable the breadth of the available literature to be covered when conducting searches for a scoping review [22]. Search terms were developed based on a small-scale preliminary search of databases and identifying commonly used language in the UK in relation to suicide risk assessment and the pre-existing literature examining the assessments. Search terms were as follows: (“suicide risk assessments" OR “screening for suicide” OR “suicide risk”) AND ("guidelines" OR “guidance” OR “advice” OR “recommendation” OR “information” OR “instruction” OR “procedure” OR “practice” OR “training”). Articles that referred to self-harm in the title and met all other inclusion criteria were included for abstract screening as the term is sometimes used to describe attempted suicide. Reference lists of included articles were hand searched for additional articles.

### Eligibility and article screening

After abstract screening, eligible articles were subjected to full-text screening. Articles were eligible for inclusion if they were original research exploring how suicide risk is assessed in the UK. Articles needed to be written in English and relate to administering or undergoing risk assessment for suicide, key concepts relating to those experiences or directly exploring the experiences of administering or undergoing assessment. Articles reporting studies using quantitative, qualitative, or mixed methods designs, were eligible (including cross-sectional, cohort, case control, and prospective or longitudinal designs). Reviews, discussion papers, non-research letters or editorials, and studies reporting non-UK data were excluded. Initial database searches were conducted by SF. EP and SF conducted abstract and full text screening independently. Hand reference searches were conducted by EP. Any disagreements on article inclusion or exclusion were discussed between SF, EP and RD, using the protocol to reach consensus.

### Data extraction

Data extracted from eligible articles included: author name(s), date of publication, study setting, country, sampling strategy, sample characteristics, participant demographics (age, sex, ethnicity, descriptive statistics), the study design, findings including qualitative and quantitative data pertaining to experiences of administering or undergoing assessment for suicide risk, and limitations of the studies. Data were initially extracted by SF and then reviewed by EP independently. Any disagreements on article inclusion or exclusion were discussed between SF, EP and RD, using the protocol to reach consensus. Disagreements regarding inclusion between reviewers related mainly to characteristics of the study and a lack of clarity around how SRA were the subject of exploration in some articles. Having charted information from the studies including findings, a narrative account of the literature was constructed with attention given to aspects of included papers which address the research question.

## Results

**Fig 1:**
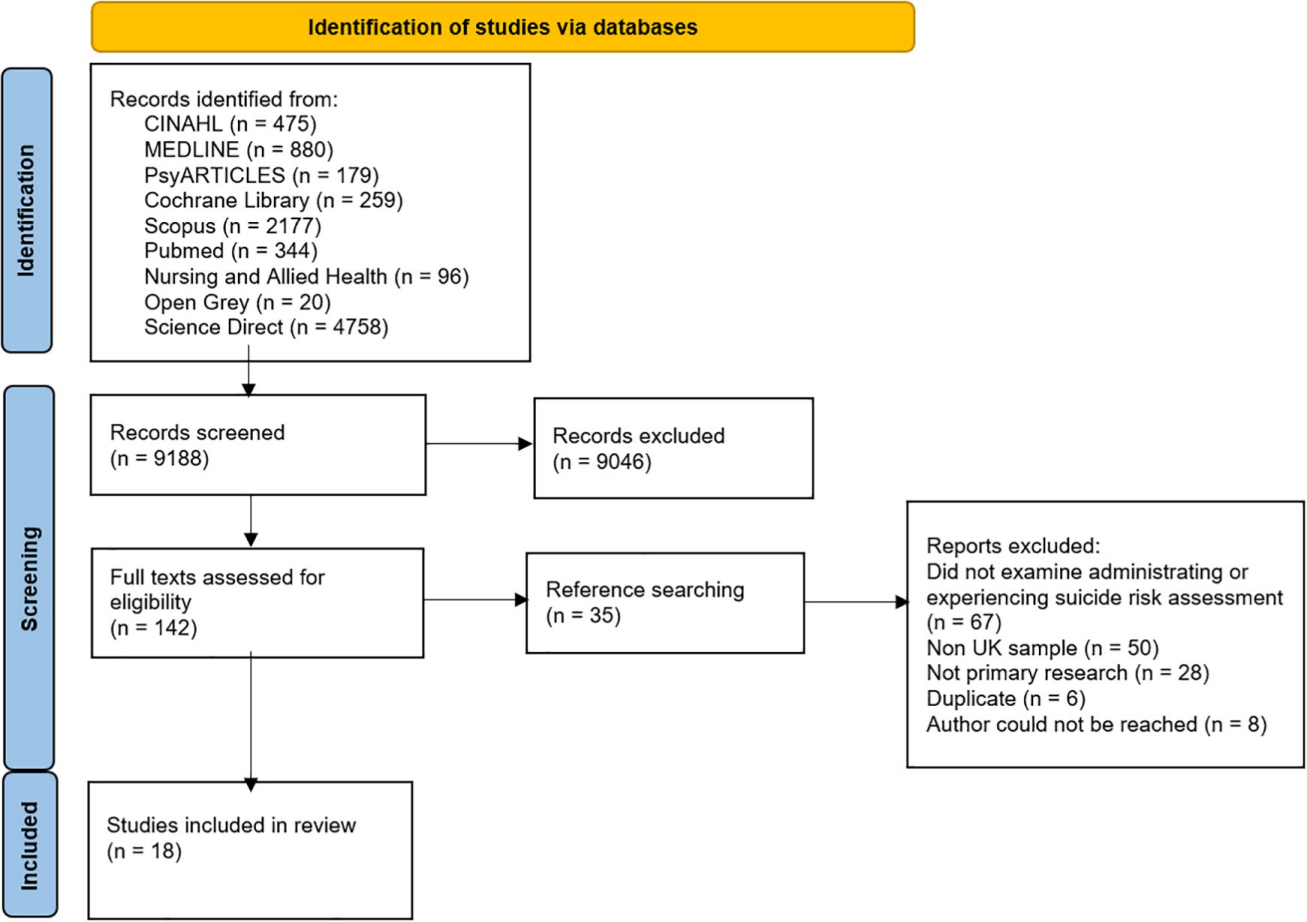
PRISMA flow chart search strategy.

As summarised in Fig 1, 9065 articles were identified in the initial search, of which 8923 were discarded following title and abstract screening. Of the full texts screened (n = 142), 126 articles were excluded (50 did not examine administrating or experiencing suicide risk assessment, 44 had a non-UK sample, 21 were not primary research, 4 were duplicates and 7 authors could not be contacted for their manuscripts), leaving 16 eligible articles. Thirty-five articles were identified from the reference list searches, of which 33 were excluded (17 did not examine administrating or experiencing suicide risk assessment, 6 had a non-UK sample, 8 were not primary research, 2 were duplicates). A final sample of 18 articles were identified for inclusion in the review.

### Characteristics of included studies

Table 1 summarises the study characteristics. Eight studies used a quantitative design [14,16,25–30]; 5 used a qualitative design [11,31–34]; and 5 used mixed methods [35–39]. All included studies were cross-sectional in design [11,14,16,25–29,31–39], except one which states a quasi-experimental controlled before and after design [30].

**Table 1:** Results of included articles.

			Sample				
Author/year	Country	Setting	Total (n)	Sample characteristics	Design/ Method	Aim	Main Findings
**Bajaj, Borreani, Ghosh, Methuen, Patel & Crawford (2008) [31]**	England	General practices in North London	204	101 patients 103 GPs	Mixed methods, cross-sectional, survey and semi-structured interviews	To examine GP and patient attitudes to screening for suicidal ideation and behaviour	• Most GPs (60.2%) had not received any formal training in how to assess risk in patients. • Barriers to screening for suicide include time pressures, cultural and language barriers, and concerns about the impact of asking about suicide.
**Buckingham, Adams, & Mace (2008) [35]**	UK	UK NHS trusts	46	21 psychiatric nurses, 14 psychiatrists, 3 social workers, 3 GPs, 5 psychologists	Qualitative, cross-sectional, interviews and content analysis	To understand how HCP conceptulise risk knowledge	• The assessor’s own reactions to the patient’s appearance and behaviour impacted on assessment. • How patients engage with the assessor is more important than what they said.
**Chandler, King, Burton & Platt (2016) [32]**	Scotland	General practices in different areas across Scotland	30	GPs	Qualitative. cross-sectional, semi-structured interviews	To explore GPs’ accounts of the relationship between self-harm and suicide and approaches to carrying out suicide risk assessments on patients who had self-harmed	• GP’s view suicide risk assessments as challenging and a continuing process. • GPs discussed deliberating the extent to which a patients’ self-harming practice was ‘truly’ suicidal and in need of immediate intervention.
**Davies, Amos & Appleby (2001) [25]**	England, Wales	NHS trusts in England and Wales	159	Clinical Directors	Quantitative, cross-sectional, survey	To establish how widespread training in risk assessment is in mental health services in England and Wales	• The existence of written policies varied. • Training was provided but it was not compulsory, so attendance is low due to staff being unable to take time away from their clinical duties.
**Gale, Hawley, Butler, Morton & Singhal** **(2016) [14]**	England	Mental health settings across Hertfordshire, Bedfordshire, and Essex	400	104 psychiatrists and doctors, 240 psychiatric nurses, 56 social workers	Quantitative, cross-sectional, non-randomised, cohort study	To investigate possible biases in suicide risk perception	• There was a significant bias across all conditions towards scoring vignettes at risk of suicide. • Many participants had high levels of confidence in their estimations.
**Graney, Hunt, Quinlivan, Rodway, Turnbull & Gianatsi (2020) [39]**	UK	Mental health trusts	358 survey responses, 22 clinician interviews	Survey responses: 27 patients, 26 carers, 109 nurses, 34 doctors, 48 clinical managers, 22 psychologists, 7 occupational therapists, 8 social workers, 62 other health professionals). Interviews: Psychiatrists 13, psychologists 9	Cross-sectional, survey and interviews	To determine which risk assessment tools are being used by mental health trusts in the UK and explore the views of clinicians	• Most participating mental health organisations used SRA tool scores to determine management decisions. • Participants discussed SRA tools facilitating communication, but they were time consuming and staff has inadequate training. • Patients and carers emphasized little involvement during the risk assessment process.
**Haq, Subramanyam & Agius (2010) [26]**	UK	Emergency department	25	Patients	Quantitative, cross-sectional. audit	To investigate the exploration of suicide risk and intent by emergency department doctors and determine if full mental state examinations had been conducted.	• Suicide risk factors and suicidal intent was poorly documented. • Mental state examination not found documented in all 25 cases.
**Kar & Prasad** **(2019) [27]**	England	Mental health services in Wolverhampton	63	Patients	Quantitative, cross-sectional, audit	To investigate risk categorisation by clinicians	• The presence of suicidal ideas did not influence risk categorisation significantly. • The presence of hopelessness led to a higher risk category.
**Leavey et al** **(2017) [11]**	Northern Ireland	General practices	91	19 GPs, 72 relatives and friends of people who have died by suicide	Qualitative, cross-sectional, semi-structured interviews	To examine barriers to effective identification and management of suicidal patients in primary care	• GPs lacked confidence in the recognition of suicidal patients. • Patients stated that GPs assessment of risk is grounded in the patient’s communication of intentions. • Participants discussed challenges in communicating with GPs. • Limited time is a key barrier to securing patient trust. • GPs acknowledged a lack of training. • GPs find suicide protocol a barrier to therapeutic engagement.
**McCabe, Sterno, Priebe, Barnes & Byng** **(2017) [36]**	UK	Outpatient psychiatric clinics and general practices	365	319 Patient and psychiatrist pairs, 46 Patient and primary care pairs	Mixed methods, cross-sectional, conversation analysis	To examine how HCP interview patients about suicidal ideation	• Patients were significantly more likely to say that they were not suicidal when the questions were negatively. • More than half of psychiatrists • significantly biased the patient’s response towards a no suicidal ideation response.
**McClatchey, Murray, Chouliara, Rowat & Hauge (2019) [37]**	Scotland	Emergency departments across Scotland	51	32 doctors, 10 consultants, 2 GP trainees, 1 GP, 4 nurses, 1 physician associate in emergency medicine.	Mixed methods, cross-sectional, survey and follow up interviews	To investigate current suicide risk assessment practices	• There was variation in suicide risk assessment tools. • Barriers to effective risk assessment included the time-consuming nature of completing a suicide risk assessment and little to no training in suicide risk assessment. • Some used a risk assessment to aid memory. • Participants felt that clinical judgment is the best means of making a decision in the absence of a robust suicide risk assessment tool.
**Michail & Tait** **(2016) [33]**	England	General practices in Nottingham	28	GPs	Qualitative, cross-sectional, focus groups	To explore general GP views and experiences of assessing suicidal young people	• GPs stated they found it difficult to identify warning signs accurately and to distinguish between signs of imminent suicide risk and changes in affect and behaviour they deemed to be a part of ‘normal adolescence’ or a ‘cry for help’. • GPs expressed concern about the usefulness and acceptability of risk assessment tools.
**Michail, Tait, & Churchill** **(2017) [28]**	England	General practices in Nottingham	70	GPs	Quantitative, cross-sectional, survey	To examine the expertise of GPs in assessing, suicidal young people	• Most GPs were unaware of any published guidelines (local, national, or international) on suicide prevention. • 44% of GPs felt confident in screening for risk factors, 13% did not. 35% reported confidence in using suicide risk assessment tools.
**Paterson, Dowding, Harries, Cassells, Morrison & Niven (2008) [29]**	Scotland	Psychiatric in-patient setting	63	12 psychiatrists, 51 nurses	Quantitative, cross-sectional, survey	To explore the factors that influence judgements regarding suicide risk	• Risk judgments across the same patient at two different time points were significantly different. • Psychiatrists were more likely to use patient diagnosis as a predictor of suicide than nurses.
**Paxton, MacDonald, Allott, Mitford, Proctor & Smith (2001) [30]**	England	General practice	34	GPs	Quantitative, intervention, survey	To determine whether the beliefs and practice of assessing suicide risk by GPs can be changed using a guidance manual	• Changes in GPs perception of assessing suicide risk and the role they play in suicide prevention were found in the intervention group.
**Quinlivan et al (2014) [16]**	England	Hospitals across England	6442	Patients	Quantitative, cross-sectional, audit	To investigate the use of risk assessments following self-harm	• In most hospitals there was a protocol or guideline in place for the immediate assessment of suicide risk for patients who presented with self-harm in the emergency department. • Unvalidated locally developed proformas were the most used instruments.
**Saini, While, Chantler, Windfuhr & Kapur (2014) [38]**	England	General practice	480	291 patients, 198 GPs	Quantitative, cross-sectional, audit of patient records and interviews with GPs	To examine risk assessment in primary and secondary care	• Only one in four practices had written policies regarding suicide or self-harm and one in five of those practices were unable to provide any specific information about what policies they followed. • Lack of training for suicide risk assessments in primary care.
**Xanthopoulou, Ryan, Lomas & Mccabe (2021) [34]**	England	Emergency Department	28	28 patients	Cross-sectional interviews	To explore the experiences of psychosocial assessment from the perspective of people attending emergency department with self-harm and suicidality	• Formulaic assessments characterised by checklist questions create a barrier to trust, disclosure and listening. Patients report feelings of being judged and unworthy of help. • Therapeutic conversations that were unscripted acknowledge patients distress and foster trust and disclosure.

#### Sample characteristics

Two articles did not clearly report the sample characteristics and are excluded from the following descriptive summaries [14,36]. Michail & Tait [33] and Michail et al [28] report data taken from the same sample, therefore, as the larger sample, only the sample characteristics of the latter are discussed here. In total, data were gathered from 8159 participants (Table 2). This comprised 1011 healthcare practitioners and 62 categorised as ‘other’ health professionals, 72 relatives and friends of people who have died by suicide, 11 social workers, 26 carers, and 6950 patients.

**Table 2:** Sample characteristics of included articles.

Sample Characteristic	(n)	% of sample	Studies
**Patients **	6977	85.51%	Bajaj et al [31], Haq et al [26], Kar & Prasad [27], Quinlivan et al [16], Saini et al [38], Xanthopoulou et al [34], Graney et al [37]
**GPs**	460	5.64%	Bajaj et al [31], Buckingham et al [35], Chandler et al [32], Leavey et al [11], McClatchey et al [37], Michail et al [28], Paxton et al [30], Saini et al [38]
**Nurses**	164	2.01%	Buckingham et al [35], McClatchey et al [37], Paterson et al [29], Graney et al [39]
**Clinical directors**	159	1.95%	Davies et al [25]
**Relatives and friends of people who have died by suicide**	72	0.88%	Leavey et al [11]
**Doctors**	66	0.81%	McClatchey et al [37], Graney et al [39]
**Other healthcare professionals**	62	0.76%	Graney et al [39]
**Clinical managers**	48	0.59%	Graney et al [39]
**Psychiatrists**	39	0.48%	Buckingham et al [35], Paterson et al [29], Graney et al [39]
**Psychologists**	36	0.44%	Buckingham et al [35], Graney et al [39]
**Carers**	26	0.32%	Graney et al [39]
**Psychiatric nurses**	21	0.26%	Buckingham et al [35]
**Social Workers**	11	0.13%	Buckingham et al [35], Graney et al [39]
**Consultants**	10	0.12%	McClatchey et al [37]
**Occupational therapists**	7	0.09%	Graney et al [39]
**Physician associate in emergency medicine**	1	0.01%	McClatchey et al [37]
**Total**	8159	100%	

The largest sub-sample of participants were patients (n = 6977), representing 85.51% of the total sample. Of this sub-sample, data were gathered from 6821 patients via audits of medical records [14,24,36,43], 27 patients by survey [37], and 129 patients by interview [31,34].

### Results synthesis

#### The extent of suicide risk assessments

There was variation across studies in terms of how widely SRAs were reported to be used in practice. For example, Bajaj et al reported that approximately 93% of GPs stated that they sometimes screen for suicidal ideation in distressed patients [31]. McClatchey et al [37] reported 68.6% of emergency department healthcare practitioners from 51 emergency departments across Scotland used an SRA tool and 31.4% did not. Of those who use a risk assessment tool, 51.4% stated that it was required by their employer, 37.1% stated it was not, and 11.4% did not know. Further to this, seven of the participating emergency departments had healthcare practitioners who disagreed as to whether an SRA tool was required, indicating a lack of consistency and confusion within departments [37]. Graney et al [39] found 94% of participating NHS mental health organisations used SRA tools to determine decisions about management and 39% used locally developed tools. The experiences of patients in one emergency department ranged from formulaic checklist SRAs to therapeutic conversations [34]. Haq et al [26] found suicide risk factors and suicidal intent identified in patients were poorly documented, finding that the mental state examinations conducted were not documented in all the 25 cases included in their study.

#### Approaches to suicide risk assessment

A variety of SRA tools and means for determining suicide risk were reported across studies. Unvalidated, locally developed, SRA tools and proformas were the most commonly reported means of assessing risk [16,37,39]. The SAD PERSONS scale was used in 28.1% of emergency departments making it the most widely used questionnaire scale to assess risk [16].

Some NHS mental health organisations approached suicide risk assessments through the use of a formulaic checklist style assessment which many patients noted leaving them feeling like their lives did not matter and feeling hopeless about the future [34]. Patients talked about the focus on risk and form filling resulting in them feeling judged, losing trust in services, and not feeling safe when discharged [34]. Other patients’ experiences of SRAs in NHS mental health organisations centred around a therapeutic conversation which helped patients feel listened to and they felt their distress acknowledged and reduced from the experience [34]. Healthcare practitioners tended to use patients’ past behaviour and psychiatric status to inform their SRAs, as well as their own initial reactions to the patient [35]. In another study, GPs talked about the importance of gaining an understanding of the patient’s wider life circumstance [32]. Some healthcare practitioners reported considering depression, care-setting post discharge, and suicidal ideation at last contact with primary care when conducting an SRA [38]. The practitioners’ own reactions to the patient’s appearance and behaviour were also often taken into consideration when conducting a risk assessment. For example, perceptions that a patient looked ‘dishevelled’ or ‘well kept’ would impact on the assessment of the level of risk for that patient [35]. Increased suicide risk was associated with hopelessness by practitioners due to hopelessness being a key component of depression and questions pertaining to depression are common in suicide risk assessment [27,35].

During consultations, some psychiatrists asked patients to confirm they are not suicidal by asking questions such as *“you’re not feeling suicidal are you*?*”*, and patients were significantly more likely to say agree with this negatively framed question (i.e., that they were not suicidal) [36]. Patients who respond to these types of questions with narrative answers, rather than a yes or no, are problematic for SRAs because it does not define risk in an unambiguous way which then maps to a specific risk category. Hence, the practitioner pursues a yes or no response from the patient following the narrative response [36]. Patients responded with a narrative in approximately one quarter of cases indicating that the forced choice of binary ‘yes’ or ‘no’ questions is problematic for the patient and does not encourage open discussion about their suicide-related experiences [36].

#### Perceptions of suicide risk assessment by healthcare practitioners, patients, carers, relatives and friends of people who have died by suicide

There was evidence of some misperceptions of suicide risk and concerns that asking about suicide could precipitate suicidal behaviours particularly in those at highest risk. For example, approximately 67% of suicides examined by Kar & Prasad [27] were viewed by healthcare practitioners as not being preventable. There is evidence of a belief amongst some practitioners that talking to their patients about suicide may in some way lead to them acting on suicidal feelings or thoughts, and that screening for suicide risk could put ideas of suicide in a patient’s head [31]. These concerns were particularly evident when discussing treatment of young people [28]. In addition, one quarter of the 101 patients from general practices in North London who responded to a survey examining attitudes towards screening for suicidality did not like being asked about suicide [31]. One in five of these patients also believed that talking about suicide in primary care may increase the likelihood of self-harm [31]. Patients were critical of the SRA process stating there is inconsistency between approaches taken in mental health organisations, with 44% describing risk assessments as being impersonal and feeling that their views were disregarded [39]. Thirty-three per cent were not aware a SRA tool was being used during their meeting and another 33% were not provided with information about crisis management [39]. On the other hand, 52% of patients felt like they were listened to by a healthcare practitioner and 45% of carers felt like their views were acknowledged [39].

Some practitioners considered the questions included in SRAs to be insensitive and stated that they find more appropriate ways of determining if a patient is having suicidal thoughts or feelings, although no details were provided about these alternatives [11]. Finding the most appropriate way to ask young people about suicide was identified by GPs as challenging, especially if the young person was accompanied by a care-giver whose presence can prevent the young person from being open about the extent of their suicidal thoughts and feelings [33]. Some healthcare practitioners reported using a risk assessment tool to aid their memory during consultations and to provide some structure but they did not use the scoring system to determine risk [37]. Many practitioners were aware that SRA tools are flawed [28,33,37] and many discussed that their clinical judgment is the best means of making a decision in the absence of a robust SRA tool [37]. GPs discussed relying on ‘gut feeling’ about patients, described as a mixture of intuition and experiential learning, to determine risk of suicide [32].

#### Variations between healthcare professionals

There were differences in how SRAs were conducted and how risk was determined across practitioners, for example, there was evidence that doctors are more likely to assign higher levels of risk compared to nurses [14] and psychiatrists being more likely to use patients’ mental health diagnosis as a predictor of suicide than nurses [29]. In addition to this, Paterson et al. [29] highlight that for 42% of psychiatrists, and 78% of nurses, risk judgements for the same patient case across two different time points were significantly different. Studies reporting healthcare practitioner confidence in screening for risk of suicide provided conflicting accounts. Some mental health practitioners (doctors and nurses), social workers, and GPs, had substantial confidence in their estimations [14,32], whilst other GPs had a lack of confidence in recognising suicidal thoughts and feelings in patients [11]. Elsewhere, Michail et al [28] state 44% of GPs felt confident in screening for risk factors and 35% reported confidence in using SRA tools. There was substantial variation between healthcare professionals in their confidence and level of risk assigned during SRAs.

#### Perceived barriers to effective suicide risk assessment

A significant barrier discussed by GPs when conducting an SRA was the time pressure during consultations [31], which was regarded as a key barrier to building trust with patients when trying to talk about suicide [11]. The time-consuming nature of SRAs make them difficult to complete within routine GP consultations, which are typically limited to 10 minutes [11]. GPs also identified the questions specified by SRAs as a barrier when attempting to engage therapeutically with patients [11]. The time needed to do a SRA was also discussed as a barrier by healthcare practitioners in NHS mental health organisations [39]. Cultural and language barriers were highlighted by patients who commented on the stigma that surrounds suicide in some religious and cultural contexts, and suggested that GPs should be more sensitive to this when asking about suicide [31].

Many healthcare practitioners perceived that some patients withhold information that would influence their suicide-risk categorisation [35]. Relatives of people who had died by suicide acknowledged that GPs’ assessment of risk is founded on the patient’s communication of how they are feeling and their intentions, and described their relative’s refusal to ‘open up’ to their GP [11]. Some practitioners reported finding it difficult to identify and distinguish signs of suicide risk and differentiate them from a ‘cry for help’ [32,33]. In this case, a ‘cry for help’ was viewed as a patient who is in distress but is not at risk of suicide. GPs discussed deliberating the extent to which a patient who is self-harming is truly suicidal or if they are using self-harm as a coping mechanism without the intention to die by suicide [32]. Leavey et al. [11] found that relatives of people who had died by suicide felt that GPs failed to recognise the potential significance of an unusual visit from a patient expressing feelings of depression. Some GPs also report feeling overwhelmed by the demand for sick notes and psychiatric medication and suspect that some patients express suicidal intent when they are not at risk of suicide as a means of securing these outcomes [11].

#### Suicide risk assessment guidance and training

There is a lack of guidance and training regarding suicide prevention amongst healthcare practitioners. Approximately 60% of GPs were unaware of published suicide prevention guidelines (including local, national or international guidance) [28]. Quinlivan et al. [16] reported that 28 of 32 surveyed hospitals had no protocol or guidelines in place for the immediate assessment of suicide risk. Approximately 60% of GPs in one study had not received any formal training in how to assess suicide risk in patients [31]. During research interviews, practitioners talked about receiving little to no suicide risk assessment training despite almost daily contact with patients experiencing suicidality [37]. Davies et al [25] reported that 76% of surveyed NHS trusts provided training to junior psychiatrists on suicide risk assessment and half provided this training to community psychiatric or ward nurses. This training was not compulsory and so attendance at these sessions were often low as staff were unable to take time away from their clinical duties [25]. Practitioners in Kar and Prasad’s (2019) study, however, described that better staff training in conducting SRAs and closer supervision of patients could have made suicides less likely [27].

In terms of written suicide prevention guidance, Davies et al [25] reported variations between NHS trusts in England and Wales in the existence of written policies pertaining to assessing risk of suicide in patients. In addition, Saini et al [38] reported one in four primary care practices had written policies for GPs to follow regarding SRA and it was also reported that one in five of those practices were unable to provide any specific information about what policies they currently follow. Paxton et al. [30] demonstrated that improvements in SRA practice could be brought about by a relatively short intervention such as the introduction of guidance for practitioners. There was substantial variation in the existence of suicide prevention guidance and policies, and staff training in suicide risk assessments, in the reviewed studies, with evidence of limited-to-no written risk assessment policies and low uptake of training.

## Discussion

A large proportion of individuals who die by suicide contact healthcare practitioners and local health services in the year prior to their death [3–5]. Suicide is one of the most preventable forms of death, as it is highly associated with psychological factors (i.e. the formation of intentions to take one’s own life) [40]. Therefore, there are opportunities for early intervention and the identification of high-risk individuals when they come into contact with healthcare staff and clinical services. The present scoping review aimed to examine the extent and range of evidence relating to how suicide risk assessments (SRAs) are conducted and experienced in the UK by healthcare practitioners, patients, carers, relatives, and friends of people who have died by suicide. The findings of this review allow insight into the everyday challenges in clinical practice related to predicting suicide. Suicide risk is not static, indeed ‘risk’ may fluctuate over time in terms of severity and in relation to other external influences (e.g., life stress). This and the low accuracy of risk assessment tools means assessment presents difficulties for healthcare practitioners. We identified evidence of a lack of training for practitioners around how to assess risk of suicide and variation in their knowledge of suicide prevention guidance [25,31,37]. The poor documentation of risk factors and suicidal ideation in patient records identified by Haq et al [26] may be a reflection of this inadequate training in and guidance for risk assessment and suicide prevention. A key message from this scoping review is the inconsistency across which assessments are used and how they are used by healthcare practitioners in the UK [16,37,39]. This is in contrast to NICE guidance which explicitly states ‘All staff who work with people of any age who self-harm should have training specific to their role so that they can provide care and treatment outlined in this guideline’ [12].

The limited time that many healthcare practitioners have to spend with patients presents a challenge to conducting safe, effective, and thorough suicide risk assessments. The time pressure on practitioners being a main barrier to building trust and developing effective communication with patients is consistent with findings from studies with healthcare staff working in the UK, USA, Australia, Canada, France, Germany, the Netherlands, New Zealand, Sweden and Norway, many of whom report feeling dissatisfied with the time they have for patient consultations [41]. Brief consultations are likely to have negative impacts on the provision of healthcare, undermine the effectiveness of risk assessments, and may contribute to healthcare practitioner stress [42]. However, Xanthopoulou et al (2021) identified that ‘therapeutic interactions’, acknowledging the distress of patients accessing Emergency Departments in England, supported people into feeling their life mattered and encouraged hope for the future [34]. This is in itself a type of intervention that takes place even in short time frames and may offer a more effective and safer means of supporting those in crisis compared to the widely used SRA measures and proformas commonly used in NHS trusts.

Unvalidated, locally developed, SRA tools were found to be the most widely used way of assessing risk, with the SAD PERSONS being the most widely used scale in the reviewed literature reported here [6]. This is particularly problematic because the SAD PERSONS scale has been shown to be no better than chance when predicting suicide within 6 months and should not be used in isolation [43,44]. Other unvalidated tools and healthcare practitioners’ own clinical judgement were commonly reported as being used during SRAs. Supported by NICE guidelines, there is a move towards SRAs being approached holistically, included the use of clinical judgement which is ideally informed by evidence, knowledge of risk factors, and clinicians’ own experience [12,45]. There were indications of some practitioners using clinical judgement and holistic approaches to assess risk in patients [32,35], although this was only explored in one study conducted by Xanthopoulou et al. [34].

It is problematic that some healthcare practitioners avoid asking patients about suicide through fear that it will incite patients to act on their suicidal thoughts and feelings, despite such assumptions not being supported by data [46,47]. Again, a lack of confidence may also play a role here, if practitioners feel unsure about how to respond to a patient disclosing suicidal ideation they may avoid asking questions about suicide [18]. Improving healthcare practitioner confidence with safely asking about suicidal experiences may be a target for further training, particularly considering the UK NICE clinical recommendations that practitioner training covers how to discuss suicidality and self-harm in an open way [12].

In some reviewed studies, some practitioners described deliberating over which of their patients are ‘truly suicidal’, while others commented that they felt some patients exaggerated distress to gain access to medication or a sick note [11,32]. The complex nature of suicidal intent makes assessing risk difficult in all but the very clear cases, and there are potentially lethal consequences to not getting such assessments right. Previous literature has discussed patients falsely claiming suicidal intent in order to access services and conversely, denying suicidal intent to avoid psychiatric treatment or involuntary hospitalization [48]. Patients whom healthcare practitioners suspect of exaggerating symptoms are less likely to receive treatment and most likely to present with suicidal ideation [49]. There is a misconception that a person cannot hold both suicidal ideation and a desire to live simultaneously, and consequently practitioners who approach suicide risk assessments with the dichotomy of who is or is not suicidal in mind, an approach encouraged by many SRA tools, will find the task more challenging [50].

### Evaluation of the included articles

Most of the reviewed literature in the present scoping review was quantitative by nature. There remains a lack of in-depth qualitative work that explores how patients, in particular, experience SRAs. There is an opportunity for improving the quality and delivery of these risk assessments by understanding the experiences of all key stakeholders involved in SRAs. Indeed, only two articles in the present review gathered data directly from patients [31,34]. Whilst it is important to understand healthcare practitioners’ experiences and perspectives, the existing literature presents a rather limited understanding of patient perspectives of SRAs. Understanding patient perspectives and experiences of SRAs may complement existing knowledge from practitioners’ perspectives and identify novel ways to conduct SRAs in a safe, collaborative, and patient-focused manner.

It was notable that the SRAs detailed in the included articles lacked a focus on the protective factors which may buffer against or reduce the risk of suicide. Instead, the assessments in the reviewed studies tended to focus on broad risk factors which precipitate or worsen suicidality (such as depressive symptoms). Factors such as perceived social support and life satisfaction should be taken into account when assessing a patient’s risk of suicide because these factors are known to moderate the association between depressive symptoms and suicidal ideation [51]. The presence of such resilience factors may be important for the patient in terms of living with suicidality and identifying those factors as part of an SRA could help to identify ways to support suicide prevention efforts for the patient. Furthermore, cultural factors such as nationality, ethnicity, and gender, have been found to play a substantial role in predicting suicide attempts, therefore such socio-cultural factors could be protective against suicide [52] or identify more specific risk factors for suicide. Cultural factors are not generally included in risk assessments and there is a limited understanding of protective factors and their role in determining a patient’s risk of suicide [53]. NICE guidance, however, states training for staff in carrying out assessments should include respecting and appreciating the cultural contexts of people’s lives [12].

### Strengths and limitations

To the authors’ knowledge this is the first attempt to synthesize the evidence on this topic in the UK. We conducted a systematic search of databases and abstract and full-text screening were conducted independently and checked. This review draws on a range of evidence and highlights gaps in the literature that require additional research to improve the success of healthcare practitioners’ assessment of patients’ suicide risk. There are, however, some limitations to acknowledge. We systematically searched several databases, including two grey literature databases, but it is possible that there is other unpublished work that could provide further insight into the use of SRAs in UK healthcare services. The exclusion of seven studies from the review, due to the authors not being contactable to provide additional information about their study, means that potentially important contributions to this review may not have been included in our synthesis of the literature. Based on the current review, caution needs to be taken in generalising these findings across different parts of the UK healthcare system. The majority of the included studies examined general practice and there is patchy sampling of other parts of the healthcare system in terms of suicide risk assessments.

### Clinical implications

Based on this scoping review, there are avenues for development in clinical practice in relation to assessing the risk of suicide. Firstly, it is possible to improve the experience of people being assessed for risk of suicide without requiring additional resources by taking a more therapeutic conversational approach, as discussed by Xanthopoulou et al [34]. The development of training and guidance around how to introduce the topic of suicide with patients, and talk sensitively about suicide in a timely manner, would appear to be particularly beneficial for many healthcare practitioners and may increase their confidence in conducting SRAs. Secondly, general training around applying NICE guidance for assessing risk of suicide in a holistic way, without a reliance on a SRA tool, to real life scenarios as part of clinical training would support healthcare practitioners in developing their confidence in assessing risk [54]. There also is a clear case for the increased involvement of patients, carers, relatives and friends of people who have died by suicide for collaborative development of care management plans in line with NICE guidance [12].

## Conclusion

This review reported the extent and range of published evidence related to how suicide risk assessments (SRAs) are conducted and experienced by healthcare practitioners, patients, carers, relatives and friends of people who have died by suicide in the UK. How these SRAs are actually used and experienced by healthcare staff (including their training in their use), particularly from the perspective of people experiencing suicidality, is not clear based on this review but may be important to study further. This review has highlighted considerable variation in the literature in terms of how SRAs are conducted in practice (e.g., the types of SRA that are used and how they are implemented), a lack of staff training and awareness of suicide prevention guidance, as well as various potential barriers to the successful use of SRAs (e.g., limited time during consultations, culture-specific considerations), including healthcare practitioner concerns about asking patients questions about suicide-related experiences. There is a need for consistency in how suicide risk is assessed across and within healthcare settings in the UK. There is also a case for a greater inclusion of the patient perspective in research exploring SRAs and how these are administered in practice. Without a more balanced and nuanced understanding of how SRAs are conducted, how they are experienced by healthcare practitioners, patients, carers, relatives and friends of people who have died by suicide, it is difficult to develop more effective means of assessing and supporting those at high risk. The early identification of those at increased risk is crucial as research indicates that a therapeutic interaction with a healthcare practitioner can reduce risk for a person experiencing suicidal thoughts and feelings by reducing distress [34]. An in-depth exploration of patient experiences of these assessments could facilitate this understanding, identify improvements to existing risk assessment tools and policies, inform more evidence-based training for healthcare practitioners, and ultimately improve the effectiveness of risk assessments for suicide.

## Supporting information

S1 Checklist PRISMA scoping review checklist. (DOCX)
